# KRAS-Mutant Non-Small Cell Lung Cancer: An Emerging Promisingly Treatable Subgroup

**DOI:** 10.3389/fonc.2021.672612

**Published:** 2021-05-03

**Authors:** Mingying Xie, Xiaoling Xu, Yun Fan

**Affiliations:** ^1^ Department of Medical Oncology, The Second Clinical Medical College of Zhejiang Chinese Medical University, Hangzhou, China; ^2^ Department of Medical Oncology, The Cancer Hospital of the University of Chinese Academy of Sciences (Zhejiang Cancer Hospital), Hangzhou, China; ^3^ Institute of Basic Medicine and Cancer (IBMC), Chinese Academy of Sciences, Hangzhou, China; ^4^ Department of Thoracic Medical Oncology, Zhejiang Cancer Hospital, Hangzhou, China

**Keywords:** KRAS-mutant, NSCLC, targeted therapy, immunotherapy, AMG510, MRTX849

## Abstract

Lung cancer, the leading cause of cancer-related deaths worldwide, can be classified into small cell lung cancer and non-small cell lung cancer (NSCLC). NSCLC is the most common histological type, accounting for 85% of all lung cancers. Kirsten rat sarcoma viral oncogene (KRAS) mutations, common in NSCLC, are associated with poor prognosis, likely due to poor responses to most systemic therapies and lack of targeted drugs. The latest published clinical trial data on new small-molecule KRAS G12C inhibitors, AMG510 and MRTX849, indicate that these molecules may potentially help treat KRAS-mutant NSCLC. Simultaneously, within the immuno-therapeutic process, immune efficacy has been observed in those patients who have *KRAS* mutations. In this article, the pathogenesis, treatment status, progress of immunotherapy, and targeted therapy of KRAS-mutant NSCLC are reviewed.

## Introduction

Lung cancer ranks first worldwide for malignant tumour-related deaths. Non-small cell lung cancer (NSCLC) is the most common histological subtype, accounting for 85% of all lung cancers (1). Compared with other mutations, Kirsten rat sarcoma viral oncogene (KRAS) mutations are among the most common mutations in NSCLC. However, patients with NSCLC harbouring KRAS mutations respond poorly to chemotherapy and have a poor overall prognosis (2). The rapid development of immunotherapy has brought hope for patients, improving the clinical outcomes of patients with KRAS-mutant NSCLC (3, 4). Currently, there are no KRAS-mutant NSCLC targeted drugs; however, promising clinical trial data on new small-molecule *KRAS* G12C inhibitors (2), AMG510 (5) and MRTX849 (6), showing that they may potentially treat KRAS-mutant NSCLC have come to light. Moreover, many different targeted drugs are currently being developed. This article summarises the current treatment options for patients with KRAS-mutant NSCLC.

## Molecular Biological Functions of KRAS

The rat sarcoma viral oncogene (*RAS*) gene mainly encodes a low molecular weight G protein (21 kD), with guanosine triphosphatase activity that acts as a molecular signal transduction switch and participates in regulating cell growth and differentiation. RAS protein is activated upon binding to guanosine triphosphate (GTP) and/or upstream signalling factors, activating downstream molecules and different signalling pathways that regulate basic cellular processes. The main RAS-mediated signalling pathways include the mitogen-activated protein kinase (MAPK) pathway (7–9); RAS-rapidly accelerated fibrosarcoma (RAF)-MAPK extracellular signal-regulated kinase (ERK) kinase (MEK)-ERK pathway, which mainly regulates cell proliferation and survival; and the phosphatidylinositol 3-kinase (PI3K)-protein kinase B (AKT)-mechanistic target of rapamycin (mTOR) pathway, which primarily controls cell proliferation. The RAS-like proto-oncogene guanine nucleotide dissociation stimulator pathway primarily stimulates the transcription of genes that promote survival and cell cycle progression. However, RAS is inactivated by guanosine diphosphate (GDP) (7). This activation/deactivation process involves GTP hydrolysis and GDP/GTP exchange, and both steps involve other regulatory proteins, such as guanine nucleotide exchange factors (GEFs) and guanosine triphosphatase activating protein (GAP).

In summary, RAS proteins regulate signal transduction by activating different effectors, thereby controlling different cellular functions.

**Figure 1:**
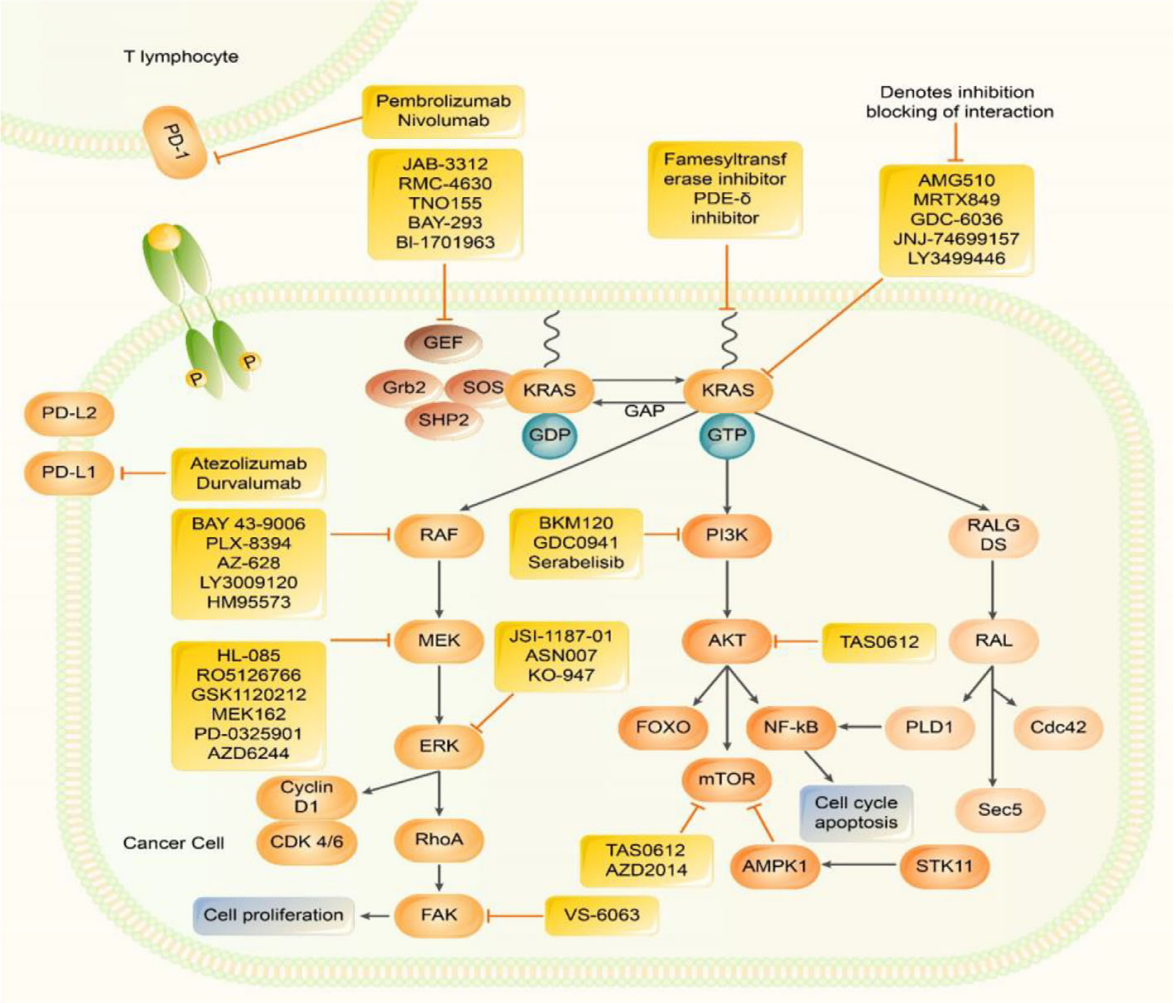
Inhibitors of Kirsten rat sarcoma viral oncogene homolog (KRAS) effector signalling. RAS protein acts as a binary molecular switch in a variety of signal transduction pathways. It is active when combined with GTP, but doesn’t have activity when combined with GDP. The GDP/GTP cycle is regulated by GEFs, which can promote the formation of active RAS - GTP and GAP stimulates GTP hydrolysis and forms inactive RAS - GDP. Normal RAS can be activated by upstream signalling factors, which in turn activates multiple downstream signalling pathways, including: MAPK, pathway; PI3k - AKT - mTOR, and pathway; RALGDS pathways. MAPK pathway, PI3K, pathway and JAK-STAT pathways promote the transcription of genes related to cell proliferation, metastasis, and drug resistance. PD -1 exists on the surface of activated T cells. When it is combined with PD-L1/2, it causes a series of immunosuppressive effects. Many Several methods have been developed to directly inhibit KRAS and inhibit KRAS downstream signalling pathways. Many new treatment strategies for KRAS inhibitors, KRAS downstream signalling pathway inhibitors, and ICIimmune checkpoint inhibitors are under investigation.

There are three genes related to human tumours in the *RAS* gene family, Harvey rat sarcoma viral oncogene (*HRAS*), *KRAS*, and Neuroblastoma rat sarcoma viral oncogene (*NRAS*), which are located on chromosomes 11, 12, and 1, respectively (10). Among them, *KRAS* most significantly impacts human cancer (**Figure 1**). The small G protein encoded by the mutated *KRAS* oncogene can still bind to GTP but prevents the GAP from increasing guanosine triphosphatase activity, inhibiting GTP hydrolysis to GDP and facilitating KRAS binding to GTP to maintain the active state. Without extracellular signals, an intracellular cascade reaction is initiated, resulting in unlimited cell growth and inducing tumourigenesis (7).

## KRAS Mutations and Their Role in NSCLC

KRAS mutations are some of the most common drivers of NSCLC and are almost only detected in lung adenocarcinoma and rarely found in squamous cell carcinoma. Over 80% of KRAS mutations occur in codon 12, and the most common mutations are *KRAS* G12C (mutation of glycine to cysteine; approximately 40%), *KRAS* G12V (mutation of glycine to valine; approximately 18–21%), and *KRAS* G12D (mutation of glycine to aspartic acid; approximately 17–18%), amongst others. Unlike other mutation types, *KRAS* mutations are mostly associated with smoking habits; approximately only 5% of *KRAS* mutations occur in light- or non-smokers. Notably, non-smokers are more likely to have *KRAS* G>A transformation mutations (mainly G12D) than smokers, while the most common mutation in smokers is a G>T translocation mutation (1, 7). Patients with KRAS-mutant NSCLC have a shorter median overall survival (OS) and a lower two-year survival rate (1).

## Prognosis of KRAS-Mutant NSCLC

At present, for NSCLC patients harbouring KRAS mutations, platinum-containing chemotherapy is central to a variety of treatments. However, the use of *KRAS* mutations as predictive markers for the onset of chemotherapy is disputable. A variety of studies have shown that *KRAS* mutations adversely affect OS and progression-free survival (PFS) and lower disease control rate (DCR) in patients with advanced NSCLC (11, 12). Furthermore, an earlier study showed that patients carrying KRAS mutations had high frequencies of liver (P = 0.01) and brain (P = 0.04) metastasis at baseline by radiological evaluation, suggesting that the presence of KRAS mutations may lead to more aggressive disease manifestations (11). As the epidermal growth factor receptor (*EGFR*) gene is located upstream of *KRAS*, a decrease in the tyrosine kinase activity of these receptors can reduce KRAS activation. However, *KRAS* mutations can counteract the therapeutic effects of EGFR tyrosine kinase inhibitors (TKIs), such as gefitinib and erlotinib, which are approved for the treatment of EGFR-mutant NSCLC but have poor efficacy in KRAS-mutant NSCLC (13).

In summary, currently available therapeutic options have little, if any, effect on NSCLC patients carrying KRAS mutations, whose prognoses remain poor.

## Progress in Immunotherapy of KRAS-Mutant NSCLC

In recent years, immunotherapy based on immune checkpoint inhibitors has been successful in treating NSCLC, especially in patients with a high tumour mutation burden (TMB), CD8^+^ tumour cell infiltration, and programmed death-ligand 1 (PD-L1) expression (14). In a retrospective study, Valero et al. showed that neutrophil-to-lymphocyte ratio (NLR) is a suitable and promising biomarker for immunotherapy (15). They suggested that higher NLR is associated with poor prognosis after immune checkpoint inhibitors (ICI) therapy. In addition, lymphocyte-to-monocyte ratio (LMR) is also associated with immune responses (16). In contrast to the NLR, the higher the LMR, the better the immune effect (16). It was recently discovered that a normal expression of the human leukocyte antigen (HLA) class is a marker of favourable responses to immunosuppressive agents/immunoinhibitors. Patients with complete loss respond poorly compared to patients with partial loss or normal expression of HLA class I (17). A recent study showed that in NSCLC, *KRAS* mutation status positively correlated with TMB, PD-L1 expression, and T cell infiltration (14). Since KRAS-mutant NSCLC is smoking-related lung cancer, high T cell infiltration and high TMB are usually observed in smokers with *KRAS* mutations (18, 19). The high T cell infiltration suggests that KRAS-mutant NSCLC may respond well to immunotherapy.

However, the influence of *KRAS* mutation status on the immune responses of NSCLC patients remains controversial. In a study of multi-line nivolumab treatment in those patients who have KRAS-mutant NSCLC (20), regardless of KRAS status, there were similar remission rates: overall response rate ([ORR] 20% vs. 17%; P = 0.39), DCR (47% vs. 41%; P = 0.23), median PFS (4 months vs. 3 months; P = 0.5), and OS (11.2 months vs. 10 months; P = 0.8). However, compared with the KRAS wild-type, the three-month PFS rate of patients with KRAS-mutant NSCLC was significantly increased (53% vs. 42%; P = 0.01). A subgroup analysis of randomised phase III study CheckMate057 indicated that during the second-line treatment for patients who carry *KRAS* mutations, nivolumab monotherapy had a higher OS benefit than docetaxel monotherapy (HR = 0.52; 95% CI: 0.29–0.95). Additionally, the subgroup with *KRAS* mutations had the highest OS benefit in nivolumab monotherapy, while the OS benefit of patients with wild-type KRAS was limited (HR = 0.98; 95% CI: 0.29–0.95) (4). According to the KRAS mutation status, the results of OS analysis in the OAK research, a randomised, double-blind III period clinical study, showed that patients with KRAS-mutant NSCLC could also benefit from atezolizumab treatment in terms of OS (HR = 0.71; 95% CI: 0.38–1.35) (3). First-line studies using immunosuppressants indicate some benefits for patients with KRAS-mutant NSCLC. The exploratory analysis of KEYNOTE-042 showed that the first-line pembrolizumab monotherapy in patients with KRAS-mutant NSCLC has higher PFS (12 months vs. 6 months; HR = 0.51; 95% CI: 0.29-0.87) and OS (28 months vs. 11 months; HR = 0.42; 95% CI: 0.22-0.81) than platinum-containing chemotherapy (21). The subgroup analysis of KEYNOTE-189 (21) showed that first-line pembrolizumab combined with platinum-containing chemotherapy has improved clinical efficacy compared with platinum-containing chemotherapy alone in patients with advanced NSCLC (PFS: 9 months vs. 5 months; HR = 0.47; 95% CI: 0.29-0.77; OS: 21 months vs. 14 months; HR = 0.79; 95% CI: 0.45-1.38). However, regardless of the *KRAS* mutation status, the PFS (9 months vs. 9 months), OS (21 months vs. 23 months), and ORR (40.7% vs. 47.6%) benefits are similar in pembrolizumab combined with platinum-containing chemotherapy. Furthermore, many studies have suggested that there may be a synergistic effect between KRAS G12C inhibitors and immunotherapy drugs. In preclinical studies, the use of AMG510 in immune-competent mice allowed T cells, especially CD8^+^ T cells, to infiltrate a large number of tumours, resulting in a pro-inflammatory tumour microenvironment that produced durable responses alone or in combination with ICI (22). Another study also verified the immunomodulatory effect of KRAS G12C inhibitors, that is, the ability to reshape the immune microenvironment (23). Therefore, the combination therapy model using KRAS G12C inhibitors and anti-PD-1 therapy is expected to become a new treatment direction.

Interestingly, the different *KRAS* mutation subtypes may be related to the immune responses of patients with NSCLC. A retrospective study suggested that (24), among the common subtypes of *KRAS* mutations, the *KRAS* G12D mutation was related to poor OS (HR: 2.43; 95% CI: 1.15–5.16; P = 0.021), while the remaining *KRAS* mutation subtypes had no significant correlation with OS. This indicates that for patients with KRAS-mutant NSCLC, the *KRAS* G12D mutation is a negative prognostic factor compared to the negative expression of PD-L1 (<1%). Additionally, *KRAS* G12C mutation is related to weakly positive expression of PD-L1 (1%–49%) which suggests that it may predict immunotherapy benefits. Another retrospective study of patients with advanced KRAS-mutant NSCLC treated with immunosuppressive agents showed no significant differences in OS or PFS among the main *KRAS* mutation subtypes (G12A, G12C, G12D, G12V, and G13C) (25).

Furthermore, *KRAS* may have co-mutations with other master genes, which may affect immunity. In one Lung Cancer Mutation Consortium (LCMC) study (1), 27% of patients with lung adenocarcinoma had *KRAS* mutations, and as many as one-third of these patients had another carcinogenic driver. Skoulidis et al. (26) discovered three clusters based on strong expression: co-mutation with serine/threonine kinase 11 (*STK11*)/liver kinase B1 (*LKB1*) known as the KL subgroup, tumour protein 53 (TP53)(KP subgroup), and cyclin-dependent kinase inhibitor 2A/B (CDNK2A/B) inactivation plus thyroid transcription factor-1 low expression (KC subgroup). In addition, Kelch-like ECH-associated protein 1 (*KEAP1*)/Nuclear factor E2 related factor 2 (*NFE2L2*) is also a critical co-mutation, which is also enriched in the KL subgroup (2, 7). These clusters had various biological characteristics and treatment reaction: the ORR of immunotherapy for NSCLC due to *KRAS* mutations alone, KRAS co-mutations with STK11/LKB1 and TP53 was approximately 28.6%, 7.4% (because the blocking of PD-1 by immunosuppressive agents was reduced), and 35.7% (showing better efficacy), respectively (27). Patients with co-mutations in KEAP1/NFE2L2 have a significantly shorter survival (HR = 1.96; 95%CI: 1.33–2.92; p ≤ 0.001). This may be owing to the high levels of tumour-infiltrating cytotoxic CD8^+^ cells, a significantly high overall mutation load, and high expression of PD-L1 in the KP subgroup tumours. In the KL subgroup tumours, *STK11* deletion promotes neutrophil recruitment, and the production of pro-inflammatory cytokines leads to a significant reduction in the number and function of T cells. Besides, *STK11*/*LKB1* inactivation reduces the expression levels of PD-L1 (24, 26, 28). KRAS-mutant NSCLC with *KEAP1* mutations were mostly immune inert tumours, with low T cell inflammation and low expression of PD-L1 ligands (7).

Blocking the PD-1/PD-L1 pathway is a promising treatment strategy for the treatment of KRAS-mutant NSCLC. Compared with immune combination chemotherapy, immunomonotherapy offers more evident PFS, ORR, and OS benefits (3, 4, 20, 21).

Cytotoxic T lymphocyte antigen-4 (CTLA-4) is considered another critical immune checkpoint, negatively regulating T cell immune responses. Ipilimumab (one of the CTLA-4 inhibitors) was widely used against melanoma (29). However, researchers are still studying the effect of CTLA-4 inhibitors on NSCLC. According to the data published by the CheckMate 227 (ClinicalTrials.gov Identifier: NCT02477826) and CheckMate 9LA (ClinicalTrials.gov Identifier: NCT03215706) studies, which included KRAS-mutant NSCLC patients, first-line treatment with nivolumab plus ipilimumab led to better survival than did chemotherapy in patients with NSCLC, regardless of PD-L1 expression level (30, 31).

In addition, tumour lymphoid-infiltrating cells are significantly cytotoxic and can accurately identify cancer cells. Therefore, therapy with these cells may be a promising new strategy.

Immunotherapy is a potential first-line treatment option for patients with KRAS-mutant NSCLC. However, because of the heterogeneity of KRAS-mutant NSCLC, especially the existence of co-mutations, individualised immunotherapy is needed.

## Progress in Targeted Therapy for KRAS-Mutant NSCLC

KRAS protein lacks a suitable “pocket” for small-molecule binding. Notably, KRAS has a very strong affinity for GTP and GDP (1000 times stronger than adenosine triphosphate [ATP]). There was very little difference between KRAS wild-type and mutant structures. Within the G or catalytic domain sequences, KRAS proteins are reportedly highly homologous with other RAS proteins, with nearly 90% similarity (10). Drugs targeting *KRAS* mutations often affect the normal KRAS. The similar structure of KRAS mutants challenges the development of effective drugs selectively targeting mutant KRAS (32).

### Direct Inhibition of *KRAS*

The most common type of *KRAS* mutation is *KRAS* G12C. The mutant cysteine is located near a pocket (P2) in the switch II region. The P2 pocket only exists in the inactive GDP-binding conformation of KRAS, which can be used to make KRAS G12C irretrievable inhibitors (33). *KRAS* G12C allele inhibitors trap oncoproteins in an inactive state by inhibiting the reactivation of exchanged nucleotides, thereby blocking the proliferation of tumour cells that depend on the protein’s signalling pathways (34). AMG510 is a selective and irreversible small molecule targeting *KRAS* G12C with a mechanism similar to that described above. Preclinical studies have shown that AMG510 can inhibit almost all measurable ERK phosphorylations, a key downstream effector of KRAS, thereby enabling *KRAS* G12C mutant tumour mice to achieve long-lasting tumour regression (22). According to the latest data published by the CodeBreak 100 study (5), among the 59 patients who carry the *KRAS* G12C mutation in patients with advanced NSCLC undergoing multi-line therapy, a total of 19 patients had definite objective remission, with an ORR of 32.2% [95% CI: 20.62–45.64]; 52 patients had clear disease controVS-6766l, with a DCR of 88.1% (95% CI: 77.07–95.09). The median PFS was 6.3 months, significantly improved compared with previous second/third-line treatments for NSCLC. In terms of safety, 39 cases (66.1%) of treatment-related adverse events and 11 cases (18.6%) of adverse events of grade 3 or above were reported. The latest research data of CodeBreak 100 Phase II was announced at the 21st World Lung Cancer Conference. Among 124 patients with evaluable efficacy, the ORR was 37.1%, the DCR was 80.6%, and the median PFS was 6.8 months. In terms of safety, during treatment with AMG510, no dose-limiting side effects were observed, and no treatment-related deaths occurred. These findings indicate that AMG510 is safe, causes remission and long-lasting benefits in patients with KRAS-mutant NSCLC (35). Based on the positive results from the preliminary clinical trials, FDA has granted Sotorasib (AMG510) the title of breakthrough therapy for the treatment of locally advanced or metastatic NSCLC with *KRAS* G12C mutation on December 8, 2020. The CodeBreak 101 (ClinicalTrials.gov Identifier: NCT04185883) study investigated AMG510 monotherapy and combination therapy with anti-tumour drugs, and the CodeBreak 200 (ClinicalTrials.gov Identifier: NCT04303780, **Table 1**) study compared the effects of AMG510 in second-line treatment with standard chemotherapy. These studies have entered the clinical trial phase, and the results are promising.

**Table 1:** Ongoing Clinical Trials of KRAS-Mutant Lung Cancer.

NCT number	Drug code	Properties	Study Phase	Intervention Model	Allocation	Blind	Sponsor
NCT04625647	AMG 510	*KRAS* G12C inhibitor	Phase 2	Single Group Assignment: AMG 510 monotherapy	Not Applicable	None	Southwest Oncology Group National Cancer Institute (NCI)
NCT04620330	VS-6766	RAF/MEK inhibitor	Phase 2	Single Group Assignment: VS-6766 monotherapy or VS-6766 in combination with defactinib	Randomised	None	Verastem, Inc.
NCT04613596	MRTX849	*KRAS* G12C inhibitor	Phase 2	Single Group Assignment: MRTX849 in combination with Pembrolizumab	Not Applicable	None	Mirati Therapeutics Inc.
NCT04470674	Durvalumab	Anti-PD-L1	Phase 2	Parallel Assignment: Durvalumab monotherapy vs Durvalumab plus chemotherapy	Randomised	None	Shirish M Gadgeel AstraZeneca Henry Ford Health System Hoosier Cancer Research Network
NCT03808558	TVB-2640	FASN inhibitor	Phase 2	Single Group Assignment: TVB-2640 monotherapy	Not Applicable	None	David E Gerber Universityof Texas Southwestern Medical Center
NCT03777124	SHR-1210; YN968D1	Anti-PD-1 antibody; VEGFR inhibitor	Phase 2	Parallel Assignment: SHR-1210 combination with apatinib vs Pemetrexed and Carboplatin	Randomised	Blind	Jiangsu HengRui Medicine Co., Ltd. Shanghai Chest Hospital
NCT03693326	PDR001	Anti-PD-1 antibody	Phase 2	Single Group Assignment: PDR001 monotherapy	Not Applicable	None	Asan Medical Center
NCT03520842	CL-14377; BAY 73-4506	antimetabolite and antifolate agent; kinase inhibitor	Phase 2	Single Group Assignment: Regorafenib in combination with Methotrexate	Not Applicable	None	Stanford University
NCT02642042	GSK1120212	MEK Inhibitor	Phase 2	Single Group Assignment: Trametinib in combination with Docetaxel	Not Applicable	None	National Cancer Institute (NCI)
NCT04303780	AMG 510	*KRAS* G12C inhibitor	Phase 3	Parallel Assignment: AMG 510 vs Docetaxel	Randomised	None	Amgen
NCT02743923	carboplatin-paclitaxel- bevacizumab; cisplatin-pemetrexed	chemotherapy	Phase 3	Parallel Assignment: carboplatin-paclitaxel- bevacizumab vs cisplatin-pemetrexed	Randomised	None	The Netherlands Cancer Institute Dutch Society of Physicians for Pulmonology and Tuberculosis
NCT02152631	LY2835219	CDK4/6 inhibitor	Phase 3	Parallel Assignment: LY2835219 vs Erlotinib	Randomised	None	Eli Lilly and Company
NCT01933932	AZD6244	MEK inhibitor	Phase 3	Parallel Assignment: Selumetinib in combination with Docetaxel vs Placebo in combination with Docetaxel	Randomised	Blind	AstraZeneca

A preclinical study of another oral, selective, small molecule (MRTX849) targeting *KRAS* G12C showed a broad spectrum of activity in tumours with the *KRAS* G12C mutation in *in vivo* models, resulting in significant tumour regression in most models (23). Mirati reported the latest clinical trial results of the Phase I/II clinical study of MRTX849 (6). In patients with advanced NSCLC who received chemotherapy and a PD-1/PD-L1 inhibitor, MRTX849 monotherapy has indicated up to 96% ORR and 45% DCR. Seventy percent (16/23) of patients with confirmed remission had more than 40% tumour reduction in comparison with the baseline. In terms of safety, 30% of patients experienced grade 3 or 4 treatment-related adverse events, 4.5% terminated treatment due to adverse reactions, and two patients died from treatment-related adverse events (one pneumonia and one heart failure case). The currently published data show that the efficacy of MRTX849 is slightly better than that of AMG510; however, the adverse effects are more significant, especially cardiac toxicity. The evaluation of the efficacy of these two drugs needs a larger cohort size. The Phase I/II clinical study (ClinicalTrials.gov Identifier: NCT04330664) of MRTX849 combined with the Src homology phosphortyrosyl phosphatase 2 (SHP2) inhibitors, TNO155, is underway.

Furthermore, the small-molecule *KRAS* G12C inhibitor JNJ-74699157 (ARS-3248) is in Phase I clinical trials (ClinicalTrials.gov Identifier: NCT04006301). Another newly developed small-molecule inhibitor, LY3499446, is under Phase I/II clinical study (ClinicalTrials.gov Identifier: NCT04165031) in combination with cyclin-dependent kinase (CDK) 4/6 inhibitor (abemaciclib), EGFR inhibitor (cetuximab), erlotinib, and docetaxel, respectively. A new *KRAS* G12C irreversible covalent inhibitor, GDC-6036, has also entered clinical trials (ClinicalTrials.gov Identifier: NCT04449874).

Although the emergence of KRAS G12C inhibitors has brought hope to patients with KRAS G12C mutations, the duration of response (DOR) (range 1.1 to 13.6 months) is not as good as the EGFR inhibitors (range 7.3 to 22.0 months) (35–37).

Furthermore, Mei Zeng et al. (38) have designed a library of C12 directed covalent degradation molecules (PROTACs). Although the degradants they found, in the end, cannot degrade the endogenous KRAS G12C, it provides new ideas and insights for the development of KRAS degradants.

In addition to the *KRAS* G12C mutation, mutations such as *KRAS* G12D also play an important role in the occurrence and development of tumours. The *KRAS* G12D-specific inhibitor MRTX1133, developed by Mirati, can reversibly bind to the activated and inactivated *KRAS* G12D mutants and inhibit their activity. The specificity of MRTX1133 to *KRAS* G12D is more than 1000 times that of wild-type *KRAS*, and its half-life is more than 50 hours (6). *In vitro* experiments indicated that MRTX1133 has a dose-dependent inhibition of the KRAS signalling pathway activity and significantly reduced the size of tumours with *KRAS* G12D mutations in pancreatic and colorectal cancer models compared with the control group (6).

### Inhibition of the Nucleotide Exchange Cycles

The conversion of inactive KRAS-GDP to active KRAS-GTP requires GEFs, including the most common one, the Son of Sevenless (SOS) protein (39). A study (40) screened out a specific small-molecule SOS1 inhibitor, BAY-293, which can effectively destroy the mutual effect between KRAS and SOS1, prevent the formation of the KRAS-SOS1 complex, and thereby inhibit the activity of all *KRAS* mutants. Another SOS1 inhibitor, BI-1701963, is in Phase I clinical trial (ClinicalTrials.gov Identifier: NCT04111458) (41).

### Inhibition of KRAS Membrane Positioning

KRAS needs to be processed by post-translational enzymes to bind to cell membranes and exert its activity, which requires the regulation of a variety of enzymes, such as farnesyltransferase, geranylgeranyltransferase, RAS-converting enzyme 1, isoprenylcysteine carboxyl methyltransferase, etc. The rate-limiting step in this series of enzymatic reactions is the isoprenylation of cysteine in the cysteine–aliphatic–aliphatic–terminal amino acid (CAAX) tetrapeptide structure mediated by farnesyltransferase (42). However, farnesyltransferase inhibitors (FTIs) such as tipifarnib, lonafarnib, and second-generation salirasib, did not show significant efficacy. This may be because when KRAS-mutant cells are deactivated by FTIs, farnesylation is deactivated. However, KRAS is modified by γ-glutamyl transpeptidase (GGT), a geranylgeranyl KRAS which allows its membrane positioning and signal transduction and overcomes the influence of FTIs (42). Simultaneous inhibition of farnesyltransferase and geranylgeranyltransferase may be an effective method, but it is necessary to observe toxicity levels. Another method to prevent the compensation effect of geranylgeranyltransferase on FTIs in KRAS-mutant NSCLC is to target other enzymes such as RAS-converting enzyme 1 and isoprenylcysteine carboxyl methyltransferase, whose inhibitors still need to be further studied. Phosphodiesterase-δ (PDE-δ) is an isoprene-binding protein that regulates the correct positioning and signal transmission of farnesylated KRAS. PDE-δ inhibitors interfere with the binding of mammalian PDE-δ and KRAS, change their location on the membrane, and inhibit carcinogenic KRAS signals (43). However, PDE-δ inhibitors’ stability is unclear, and they may lack sufficient selectivity for KRAS protein, thus, warranting further research.

### Inhibition of the Downstream Signal Pathway of KRAS

RAF-MEK-ERK pathway inhibition: Sorafenib (BAY 43-9006) is the first compound developed specifically for RAF. It is a multi-TKI (not a specific RAF kinase inhibitor) against vascular EGFR, platelet-derived growth factor receptor, and proto-oncogene tyrosine-protein kinase (44). In the BATTLE trial and the phase III MISSION trial, sorafenib did not have a noticeable therapeutic effect on KRAS-mutant NSCLC, nor did it prove *KRAS*-mutant state has predictive value for the efficacy of sorafenib (45–47). Unlike sorafenib, v-RAF murine sarcoma viral oncogene homolog B1 (BRAF) inhibitors are RAF-specific inhibitors. Currently, many BRAF inhibitors, for instance, dabrafenib, vemurafenib, and encorafenib, have been approved to target *BRAF* V600 mutations, but RAF kinase inhibitors do not perform well in *KRAS*-mutant cells (48, 49). ATP-competitive RAF inhibitors inhibit ERK signalling in mutant *BRAF* cells but enhance signal transduction in wild-type *BRAF* cells (50). The study found that type 1.5 RAF inhibitor, PLX-8394, and type II inhibitors, AZ-628 and LY3009120, had a certain inhibitory effect on *KRAS*-mutant cells and did not cause the contradictory MAPK pathway activation (49). An effective RAF inhibitor, HM95573 (belvarafenib), is under Phase I clinical study (ClinicalTrials.gov Identifier: NCT03284502).

MEK is a serine/threonine kinase, a downstream signal of KRAS and BRAF. Activated RAF activates MEK, which activates ERK and other transcription factors, in turn promoting cell cycle progression and cell proliferation. MEK inhibitors have shown potential efficacy in cancers with MEK or BRAF mutations, especially in BRAF V600E mutant tumour cell lines (50, 51). However, data from a number of studies have shown that the MEK1/MEK2 inhibitors, smeltinib (AZD6244; ARRY-142886) and trametinib (GSKll20212) cannot improve the prognosis of patients with KRAS-mutant NSCLC (52, 53). The reason may be that MEK inhibitors can induce signal feedback of the MAPK pathway in *KRAS*-mutant tumours, resulting in drug resistance to MEK inhibitors (54). HL-085 is a new ATP non-competitive MEK inhibitor in Phase I clinical trial (ClinicalTrials.gov Identifier: NCT03990077). Binimetinib (MEK162) is a selective MEK1/2 inhibitor in ongoing clinical trials (ClinicalTrials.gov Identifier: NCT01859026 and NCT02964689). Another Phase I clinical trial (ClinicalTrials.gov Identifier: NCT01986166) of the combination of the MEK inhibitor cobimetinib (GDC-0973) with MEHD7945A has not yet announced its results. Hyejin Choi et al. (55) used MEK inhibitors (MEKis) for pulsatile treatment in preclinical studies instead of continuous treatment. They found that the pulse regimen alone has a better anti-tumour effect and delayed the emergence of drug resistance. In addition, pulse MEK treatment combined with CTLA-4 blockade can prolong the survival time of KRAS-mutant tumour in mice, which may be related to T cell activation and increased CTLA-4 expression due to MEK pulse therapy.

A single application of a MEK- or RAF inhibitor for *KRAS* mutations shows no clinical efficacy. On the contrary, the combined application of a MEK inhibitor and a RAF inhibitor may be a feasible strategy. Combining the RAF inhibitor LXH254 and the MEK inhibitor trametinib is currently in Phase I clinical trial (ClinicalTrials.gov Identifier: NCT02974725). A Phase I clinical study (ClinicalTrials.gov Identifier: NCT03284502) of belvarafenib combined with cobimetinib is in progress. VS-6766 (RO5126766), a new targeted drug, whose Phase II clinical study (ClinicalTrials.gov Identifier: NCT04620330) is underway, inhibits both MEK and RAF.

SHP2 plays an indispensable role in *KRAS* mutation-driven tumours. SHP2 is involved in the downstream signal transduction of a variety of growth factors, cytokines, and integrin receptors, and its reduced activity inhibits tumour progression (56). Ruess et al. (56) reported that the combination of SHP2 with MEK inhibitors to target the xenograft models of KRAS-mutant NSCLC resulted in a synergistic effect to control tumour growth continuously. The RMC-4630 single drug Phase I clinical study (ClinicalTrials.gov Identifier: NCT03634982) and the clinical trial (ClinicalTrials.gov Identifier: NCT04418661) on its combination with pembrolizumab have been launched. TNO155 has also entered a Phase I clinical trial (ClinicalTrials.gov Identifier: NCT03114319). Notably, JAB-3312 can block the PD-1 pathway of T cells and the KRAS-MAPK pathway of tumour cells by inhibiting SHP2; thus, it plays a dual role in tumour immunity and tumour targeting. It is currently in Phase I clinical studies both in China and abroad (ClinicalTrials.gov Identifier: NCT04121286 and NCT04045496).

ERK is the final kinase in the MAPK pathway. The resistance of *KRAS*-mutated tumours to RAF or MEK inhibitors is usually caused by ERK feedback activation. Combined inhibition of ERK may be a feasible strategy to prevent drug resistance. Currently, ERK inhibitors such as JSI-1187-01, ASN007, and KO-947 are in Phase I clinical trials; ClinicalTrials.gov Identifier: NCT04418167, NCT03415126, and NCT03051035, respectively.

PI3K-AKT-mTOR pathway inhibition: PI3K is a cell effector molecule downstream of KRAS, and PI3K inhibitors BKM120, GDC0941, and XL147 have shown promising results in Phase I clinical trials (57–59). Serabelisib is a P13K catalytic subunit inhibitor and is in a Phase I/II clinical study (ClinicalTrials.gov Identifier: NCT04073680). TAS0612 is a new AKT inhibitor in Phase I clinical trial (ClinicalTrials.gov Identifier: NCT04586270). mTOR is a serine/threonine kinase downstream of PI3K in the PI3K-AKT-mTOR pathway. The mTOR inhibitor rapamycin and its analogues (CCI-779, RAD001, and AP23573), which induce cell cycle arrest in the G1 Phase, have certain anti-tumour activity in NSCLC (60). AZD2014 is a new mTOR inhibitor in Phase I/II clinical studies (ClinicalTrials.gov Identifier: NCT02583542).

Inactivation of a single MAPK or PI3K pathway has poor efficacy in *KRAS*-mutated tumours. The inhibition of the MAPK pathway activates the PI3K pathway, reducing *KRAS*-mutated cell sensitivity to MEK inhibitors (61). Therefore, the P13K-AKT-mTOR and RAF-MEK-ERK pathways were targeted simultaneously may be a promising strategy, but its toxicity should be observed.

Janus kinase-signal transducer and activator of transcription 3 (STAT3) inhibition: In KRAS-mutant NSCLC, after inhibiting MEK, STAT3 is activated *via* fibroblast growth factor receptor and Janus kinase; combined inhibition of this receptor, MEK, and Janus kinase can promote tumour regression (62).

### Inhibition of *KRAS* Synergetic Genes

*KRAS*-mutated tumour cells can be killed by inhibiting other synergetic lethal genes responsible for their growth and survival. There are many transcription factors, including Wilms tumour 1 and GATA (A conserved sequence in a gene promoter whose core base sequence is Cys-X2-Cys-X17-Cys-X2-Cys)-binding protein 2 (GATA2) and small molecules involved in the nuclear factor kappa B (NF-κB) pathway. Wilms tumour 1 is a key regulator of ageing and proliferation downstream of oncogenic *KRAS* signalling (63). Kumar et al. (64) demonstrated that KRAS-mutant NSCLC relies on GATA2, and the deletion of GATA2 reduces the activity of KRAS-mutant NSCLC cells without affecting the wild-type cells. Bortezomib, a proteasome inhibitor that affects ubiquitin-proteasome pathways, disrupts protein homeostasis, leads to cell cycle interruption, inhibits transcription factors such as NF-κB, and produces anti-angiogenic effects, which inhibit tumour growth and proliferation, ultimately leading to apoptosis (65). In addition, the nuclear outlet receptor, exportin 1, has a strong synergetic lethal effect on *KRAS*-mutated cancer cells *in vitro* and *in vivo* (66). The exportin 1 inhibitor selinexor (KPT-330) is currently under Phase I/II clinical study (ClinicalTrials.gov Identifier: NCT03095612).

Heat shock protein 90 (HSP90) is a conservative and highly active molecular chaperone protein that stabilises the protein conformation of important signal transduction factors in the tumour pathogenesis pathway and protects the proteasome from degradation. Sos et al. (67) found that *KRAS* mutations enhanced tumour dependence on HSP90. They also found that tumours significantly regressed when treated with HSP90 inhibitors in a mouse model of lung adenocarcinoma driven by *KRAS*. Ganetespib is an HSP90 inhibitor. In a Phase II study, ganetespib monotherapy showed efficacy in KRAS-mutant NSCLC, but it was more significant in patients with anaplastic lymphoma kinase fusion (68). In a Phase II trial of ganetespib combined with docetaxel, the combination failed to improve PFS or OS in patients with KRAS-mutant NSCLC (69). AUY922 is a highly effective ATP-competitive HSP90 inhibitor. Although preclinical research results have shown that KRAS-mutant NSCLC is sensitive to AUY922, no clinical benefit of AUY922 has been observed in patients with *KRAS* mutations (70). Puyol et al. (71) found that CDK4 has a specific synthetic effect on KRAS-driven NSCLC. Abemaciclib and palbociclib (PD-0332991) are both CDK4/6 inhibitors under clinical study.

### Other Therapy Options

Mesenchymal-epithelial transition factor (MET) is a transmembrane tyrosine kinase receptor involved in invasion, proliferation, angiogenesis, and metastasis and can also activate the KRAS pathway. MET amplification is discovered from approximately 4% of lung adenocarcinomas and leads to resistance to EGFR TKIs *via* activating the KRAS-PI3K-AKT-mTOR pathway. Currently, MET inhibitors include onartuzumab, a monoclonal antibody targeting the MET receptor, and tivantinib (ARQ 197), a small-molecule c-MET receptor TKI. In a Phase II study of onartuzumab combined with erlotinib, no response was observed in KRAS-mutant NSCLC (72). Another randomised Phase II study showed (73) that tivantinib combined with erlotinib did not improve prognosis in patients with unselected advanced NSCLC (PFS was 3.8 months in the ET group and 2.3 months in the EP group, respectively (HR: 0.81; 95% CI: 0.57–1.16). However, an exploratory analysis showed a significant improvement in PFS in patients with KRAS-mutant NSCLC (HR: 0.18; 95% CI: 0.05–0.70; P = 0.006).

Focal adhesion kinase (FAK) participates in the adhesion between cells and the extracellular matrix. The ERK-RAS Homolog Family Member A (RHOA) -FAK pathway is necessary to maintain KRAS-mutant lung adenocarcinoma. Inhibition of FAK can selectively induce KRAS-mutant cell death and lead to KRAS-mutant lung cancer regression (74). In a Phase II study (ClinicalTrials.gov Identifier: NCT01778803) (75) on defactinib (VS-6063; a selective oral inhibitor of FAK) treatment of advanced KRAS-mutant NSCLC, defactinib monotherapy showed moderate clinical activity. The study included 55 patients with KRAS-mutant NSCLC; 15 patients (28%) achieved a 12-week PFS endpoint, and one patient achieved partial remission with a median PFS of 45 days. Moreover, defactinib was generally well-tolerated.

Human vascular endothelial growth factor (VEGF) plays a vital role in promoting the proliferation, migration, and survival of endothelial cells (ECs); VEGF also can stimulate tumour angiogenesis. Besides, bevacizumab can directly inhibit deoxyribonucleic acid (DNA) repair in tumour cells. The reason may be that anti-angiogenic therapy can downregulate DNA repair genes, such as excision repair cross complementary gene 1 (ERCC-1) and X-ray repair of complementary cross gene 1 (XRCC-1), thereby enhancing the radiosensitivity of tumours (76). Some studies showed that bevacizumab combined with chemotherapy had no survival benefit for KRAS-mutant NSCLC (77, 78). Poly adenosine diphosphate (ADP)-ribose polymerase 1 (PARP1) plays an essential role in DNA damage repair and apoptosis, which, combined with WEE1 inhibitors, is associated with the killing of 25%-40% of KRAS-mutant NSCLC cells (79).

Many other combined inhibition therapies are available, for example, the combined inhibition of mTOR and Wee1 nuclear kinase (80), combined inhibition of checkpoint kinase 1 and MAPK-activated protein kinase 2 (81), and MEK/Bromodomain and extraterminal combined inhibitors (82), which have shown synergistic effects in preclinical studies and need to be demonstrated in further clinical trials.

## Prospects

The treatment of lung cancer has made rapid progress due to developments in medicine, particularly immunotherapy. The immunotherapy of KRAS-mutant NSCLC has shown promising efficacy. Many studies have indicated that immunotherapy can be recommended as the first-line treatment for KRAS-mutant NSCLC. However, it is also necessary to pay attention to the existence of its mutation subtypes and co-mutations and design individualised treatment. The clinical trials on AMG510 and MRTX849, inhibitors that directly target *KRAS* G12C, have shown surprising results. Nevertheless, the efficacy, duration of efficacy, and potential drug resistance of *KRAS* G12C inhibitors in treating different mutation subtypes warrant further research. Targeting KRAS downstream effector molecules (PI3K, BRAF, mTOR, MEK, etc.), especially the combined use of downstream effector molecule inhibitors, shows promising prospects. Furthermore, the combination of a variety of therapeutic drugs with different mechanisms has shown synergistic effects in preclinical studies and is a promising strategy that can improve drug efficacy and solve drug resistance. We believe that drug combinations can help patients with KRAS-mutant NSCLC bring more effective treatment.

## Author Contributions

MX performed the literature search, wrote the manuscript, and guaranteed its integrity. YF conceived the framework of the manuscript. XX revised the entire manuscript. All authors contributed to the article and approved the submitted version.

## Funding

This work was supported by the Natural Scientific Foundation of China (Nos. 81972718), the Natural Scientific Foundation of Zhejiang Province, China (No. LY19H160007).

## Conflict of Interest

The authors declare that the research was conducted in the absence of any commercial or financial relationships that could be construed as a potential conflict of interest.
